# Evaluation of clinically relevant sevoflurane concentrations in the development of malignant hyperthermia in an *Ryr1* mutant mouse model

**DOI:** 10.1186/s12871-026-03779-y

**Published:** 2026-03-24

**Authors:** Kenshiro Kido, Hirotsugu Miyoshi, Tsuyoshi Ikeda, Guoqiang Xia, Ayako Sumii, Sachiko Otsuki, Keiko Mukaida, Yusuke Sotomaru, Satoru Noguchi, Yasuo M. Tsutsumi

**Affiliations:** 1 Department of Anesthesiology and Critical Care, Hiroshima University Hospital, 1-2-3 Kasumi, Minami-Ku, Hiroshima, 734-8551 Japan; 2 Natural Science Center for Basic Research and Development, Hiroshima University, Hiroshima, Japan; 3 Department of Neuromuscular Research, National Center of Neurology and Psychiatry (NCNP), National Institute of Neuroscience, Tokyo, Japan

**Keywords:** Malignant hyperthermia, Ryanodine receptor type 1, Sevoflurane

## Abstract

**Background:**

Malignant hyperthermia (MH) is associated with mutations in the *RYR1* gene; however, the effect of volatile anesthetic concentrations on the clinical symptoms of patients with MH is unknown. We investigated the effects of high concentrations of volatile anesthetics on MH development and progression.

**Methods:**

MH mouse models with R2509C mutation in *Ryr1*, all male, approximately 15 weeks old were administered 5%, 2.5% or 0.6% sevoflurane. The time at maximum temperature, maximum temperature, time at maximum heart rate, maximum heart rate, and time to asystole in the first experiment were measured. In the second experiment, blood samples were obtained from mice after the administration of 5% or 0.6% sevoflurane. In this experiment, the pH, base excess, and electrolyte and lactate levels were measured.

**Results:**

Among the 16 mutant mice used, all showed elevation of heart rate and body temperature and went into asystole. The 0.6% group showed longer time at maximum temperature than the 5% group (68.0 [67.0, 72.0] min vs 43.5 [18.3, 47.8] min, *p* = 0.046); while, maximum temperature was higher in the 0.6% group than the other groups (40.2 [38.2, 40.8] °C in the 5% group, 42.4 [42.2, 42.6] °C in the 2.5% group, and 44.7 [43.6, 45.3] °C in the 0.6% group); maximum heart rate was lower in the 5% group (480 [465, 503] bpm in the 5% group, 630 [570, 630] bpm in the 2.5% group, and 630 [630, 660] bpm in the 0.6% group). In the second experiment, the 5% group showed lower base excess (-23.4 [-24.4, -22.3] mmol/L vs -5.3 [-7.2, -4.3] mmol/L, *p* = 0.043) and higher lactate levels than the 0.6% group (20.00 [19.90, 20.00] mmol/L vs 4.80 [4.35, 7.88] mmol/L, *p* = 0.022). Potassium was relatively higher in the 5% group than in the 0.6% group.

**Conclusions:**

When exposed to high concentrations of sevoflurane, MH developed and progressed rapidly; however, the maximum temperature was lower than that in cases of low-concentration exposure, likely due to the rapid progression of electrolyte abnormalities.

## Introduction

Malignant hyperthermia (MH) is known as one of the most potentially fatal complications due to general anesthesia. MH is triggered by volatile anesthetics or depolarizing muscle relaxants, resulting from the excessive release of calcium from the sarcoplasmic reticulum in skeletal muscle cells [1, 2]. Clinically, MH is characterized by symptoms such as high fever, tachycardia and muscle rigidity. As the condition progresses, it can lead to severe rhabdomyolysis and lactic acidosis, which may result in life-threatening arrhythmias [3]. Although the appropriate administration of dantrolene and body cooling has improved patient outcomes, MH remains a complication with a 1 to 5% mortality rate [3–5]. Immediate treatment of MH is important to decrease mortality in patients [6], and the pathogenesis of MH in the early stages of the clinical course should be revealed. However, early symptoms of MH are diverse and non-specific, making early detection difficult. This is a major challenge in MH management.

MH is a genetic disease associated with mutations in several genes. One of the most important genes known to be associated with MH is the *RYR1* gene, which encodes ryanodine receptor type 1 [7]. Some researchers have reported on functional analysis using in vitro cells with mutations in *RYR1*, [8, 9] and various animal models, including pigs, which have also been used to study the pathogenesis of MH. Regarding MH susceptibility and management of volatile anesthetic concentrations, it is considered appropriate that patients with MH susceptibility should not be exposed to volatile anesthetics at concentrations higher than 5 ppm (0.0005%) [10]. However, it remains unknown how patients would present clinically when exposed to low concentrations of volatile anesthetics. Generally, higher exposure to stimulants leads to stronger responses. In vitro analyses have demonstrated that exposure to stimulants, such as 4-chloro-m-cresol, caffeine, or KCl, results in a concentration-dependent increase in calcium release [8, 9, 11]; however, the relationship of the differences in clinical progression when individuals with an MH predisposition are exposed to varying concentrations of volatile anesthetics remain unclear.

To address this uncertainty, we hypothesized that a high concentration of volatile anesthetics might accelerate the progression of MH, leading to higher peak body temperature. In this study, we used an MH mouse model carrying the R2509C mutation in *Ryr1* to investigate the effects of high concentrations of volatile anesthetics on MH development and progression.

## Methods

### Ethics

All animal experiments were conducted in accordance with the ethical guidelines for animal research and approved by the Hiroshima University Animal Research Committee (Approval No. A23-60–2). Recombinant DNA experiments were approved by the Hiroshima University Safety Committee for Living Modified Organisms (Approval No. 2023–107-2). The mice were housed in a controlled environment with standard food and water ad libitum, and anesthesia was administered as necessary to reduce pain and distress. Humane endpoints were established, and euthanasia was performed in accordance with institutional guidelines. When euthanasia was needed, mice were euthanized by cervical dislocation under general anesthesia with sevoflurane.

### Animals

In previous studies, our team has reported that the R2508C mutation in *RYR1* is pathogenic and related to MH in cellular experiments [8, 12]. In addition, Yamazawa et al. reported a novel RYR1-selective inhibitor using model mice with an R2509C mutation in *Ryr1*, which is equivalent to R2508C in the human *RYR1* gene, and these mice showed symptoms of MH due to isoflurane [13]. Therefore, we used the same model mice in this study. The mice were originally owned by Department of Neuromuscular Research, National Center of Neurology and Psychiatry (NCNP), National Institute of Neuroscience (Tokyo, Japan), and we obtained informed consent to use the mice in this study. We used only male mice considering sexual difference of MH incidence in human patients [3], and they were approximately 15 weeks old. Mice were housed in isolator cages, fed with food and water ad libitum, and kept in a controlled environment with 12/12 light/dark cycles, 23–25 °C temperature, and 50–60% relative humidity under specific pathogen-free conditions at the Natural Science Center for Basic Research and Development of Hiroshima University.

### Experiment 1

The mice were sedated by intraperitoneal administration of medetomidine (0.3 mg/kg), midazolam (4 mg/kg), and butorphanol (5 mg/kg). After sedation, patients were intubated and managed with mechanical ventilation. For intubation, we used 20-gauge intravenous catheters originally made for human patients and adopted pressure-controlled ventilation with an inspiratory pressure of 15 cmH_2_O and a respiratory rate of 110 bpm. After intubation, we administered 0.5 L/min of oxygen and sevoflurane with different concentrations: 5%, 2.5%, and 0.6% to the mouse models. Puig et al. previously reported that 3% of sevoflurane is equivalent to 1.2 minimum alveolar concentration (MAC) [14]; hence, 2.5% can be estimated as around 1 MAC. Randomization was not used to allocate mice to each group. The rectal temperature and heart rate were recorded for each mouse during the entire experiment. Outcome measures included the time at maximum temperature, maximum temperature, time at maximum heart rate, maximum heart rate, and time to asystole. We analyzed and compared the outcomes among the three groups.

### Experiment 2

To assess the severity of sevoflurane-induced electrolyte abnormalities, another blood test was planned. The model mice were sedated as described in the first experiment. After intubation and starting mechanical ventilation with 0.5 L/min of oxygen, sevoflurane (5% or 0.6%) was administered. After 30 min, arterial blood samples were collected via cardiac puncture. In some of the mouse models, blood samples were collected after intubation without sevoflurane administration. We examined the pH, levels of oxygen and carbon dioxide, and electrolytes, including potassium, lactate, glucose, and creatinine, for each sample and compared them among the three groups.

### Statistical analysis

Data are presented as medians and interquartile ranges. In both experiments, the Kruskal–Wallis and Steel–Dwass tests were used for statistical analysis, and differences were considered significant when the *p*-values were < 0.05. R (version 4.2.2; R Core Team, Vienna, Austria) was used for statistical analysis.

## Results

### Experiment 1

**Fig. 1:**
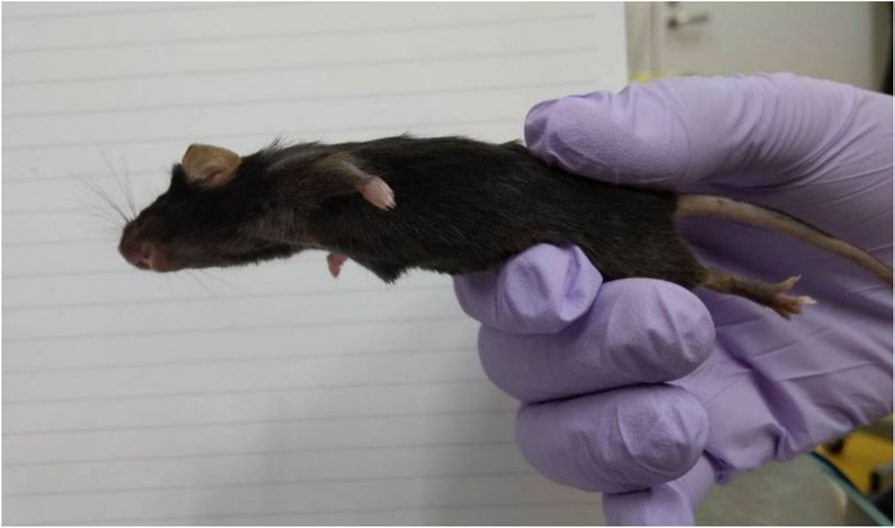
A mouse exhibiting muscle rigidity due to malignant hyperthermia. All mice exhibited MH symptoms, including fever, tachycardia, and muscle rigidity

**Fig. 2:**
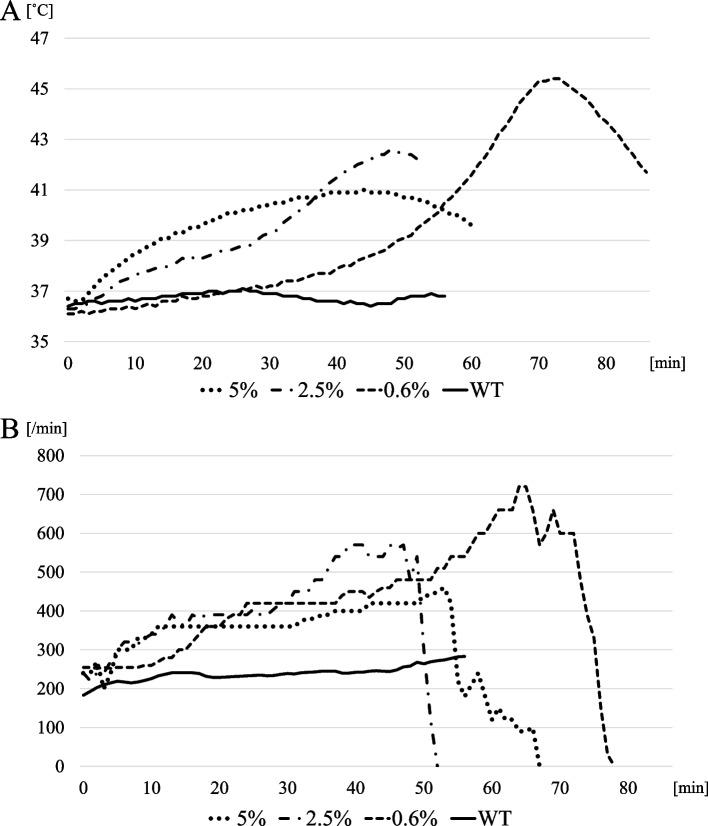
Typical changes in body temperature and heart rate in mice exposed to different concentrations of sevoflurane. Examples of the course of vital signs are shown. In each figure, the changes caused by different concentrations are represented by different types of lines. The change in vital signs of wild type mice with 2.5% sevoflurane is shown as ‘WT’. The maximum body temperature and heart rate were higher at lower concentrations. **A** Changes in body temperature of the mouse model. **B** Changes in heart rate of the mouse model

Sixteen mutant mice were used for the first experiment. Among them, six mice were administered 5% sevoflurane, five were administered 2.5% sevoflurane, and five were administered 0.6% sevoflurane. No significant differences were observed in the basic characteristics of the mouse models (Table 1). All mutant mice showed elevation of heart rate and body temperature after exposure to sevoflurane and finally went into asystole. At the end of the experiment, they showed muscle rigidity. On the other hand, we also administered wild type mice sevoflurane, but they did not show significant change in vital signs (Figs. 1 and 2).

**Table 1:** Basic characteristics of mice used in Experiment 1

	A: 5% (*n* = 6)	B: 2.5% (*n* = 5)	C: 0.6% (*n* = 5)	*P* value			
				All	A vs B	A vs C	B vs C
Age (weeks)	14 [14, 16]	15 [15]	15 [15]	0.467	0.551	0.957	0.372
Body weight (g)	31.5 [30.0, 33.1]	29.9 [25.6, 29.9]	29.0 [26.9, 29.7]	0.109	0.221	0.161	0.860
Initial body temperature (˚C)	36.5 [36.3, 36.5]	36.6 [36.3, 36.9]	36.1 [35.8, 36.1]	0.059	0.624	0.220	0.069
Initial heart rate (/min)	250 [240, 290]	240 [225, 270]	228 [210, 240]	0.276	0.740	0.259	0.678

Six mutant mice were administered 5% sevoflurane, five were administered 2.5% sevoflurane, and five were administered 0.6% sevoflurane. There were no significant differences in age, body weight, initial body temperature, or heart rate between groups. Each value is shown as the median and interquartile range

For outcomes, time at maximum temperature was significantly longer in the 0.6% group than the 5% group (68.0 [67.0, 72.0] min vs 43.5 [18.3, 47.8] min, *p* = 0.046), and time to asystole was longer in the 0.6% group than the 2.5% group (82.0 [78.0, 86.0] min vs 52.0 [50.0, 56.0] min, *p* = 0.024) (Table 2). However, maximum temperature was significantly higher in the 0.6% group than the other groups (40.2 [38.2, 40.8] °C in the 5% group, 42.4 [42.2, 42.6] °C in the 2.5% group, and 44.7 [43.6, 45.3] °C in the 0.6% group). Also, maximum heart rate was lower in the 5% group (480 [465, 503] bpm in the 5% group, 630 [570, 630] bpm in the 2.5% group, and 630 [630, 660] bpm in the 0.6% group).

**Table 2:** Outcomes for Experiment 1

	A: 5% (*n* = 6)	B: 2.5% (*n* = 5)	C: 0.6% (*n* = 5)	*P* value			
				All	A vs B	A vs C	B vs C
Time at maximum temperature (min)	43.5 [18.3, 47.8]	48.0 [37.0, 48.0]	68.0 [67.0, 72.0]	0.037	0.981	0.046	0.110
Maximum temperature (˚C)	40.2 [38.2, 40.8]	42.4 [42.2, 42.6]	44.7 [43.6, 45.3]	0.003	0.158	0.017	0.024
Time at maximum heart rate (min)	50.0 [41.0, 55.3]	42.0 [39.0, 50.0]	65.0 [64.0, 67.0]	0.077	0.510	0.406	0.072
Maximum heart rate (/min)	480 [465, 503]	630 [570, 630]	630 [630, 660]	0.003	0.016	0.016	0.460
Time to asystole (min)	65.5 [61.0, 79.0]	52.0 [50.0, 56.0]	82.0 [78.0, 86.0]	0.022	0.110	0.745	0.024

The 0.6% group showed a longer time at maximum temperature than the 5% group, and a longer time to asystole than the 2.5% group. However, the maximum temperature was significantly higher in the 0.6% group than in the other groups, while the maximum heart rate was lower in the 5% group. Each value is shown as the median and interquartile range

### Experiment 2

The results of the second experiment are listed in Table 3. Fifteen mutant mice were randomly assigned to three groups (*n* = 5 per group): 5% sevoflurane, 0.6% sevoflurane, and no sevoflurane exposure. Compared with the 0.6% group, the 5% group showed lower base excess (−23.4 [−24.4, −22.3] mmol/L vs −5.3 [−7.2, −4.3] mmol/L, *p* = 0.043) and higher level of lactate (20.00 [19.90, 20.00] mmol/L vs 4.80 [4.35, 7.88] mmol/L, *p* = 0.022). In addition, the pH, potassium, and anion gap were higher than those in mice without sevoflurane, but were not significantly different between the 5% and 0.6% groups. However, especially as for potassium, mice with 5% of sevoflurane showed relatively higher levels than mice with 0.6% (9.1 [8.6, 9.6] mmol/L vs 6.4 [5.5, 7.3] mmol/L).

**Table 3:** Results of blood tests in Experiment 2

	A: 5% (*n* = 5)	B: 0.6% (*n* = 5)	C: no sevoflurane (*n* = 5)	*P* value			
				All	A vs B	A vs C	B vs C
pH	6.821 [6.771, 6.838]	6.960 [6.922, 7.294]	7.360 [7.318, 7.535]	0.014	0.363	0.024	0.116
PaO_2_ (mmHg)	58.8 [39.9, 70.7]	64.4 [34.8, 87.6]	341.1 [209.1, 373.9]	0.336	0.994	0.363	0.484
PaCO_2_ (mmHg)	87.9 [62.1, 107.8]	110.2 [28.7, 140.5]	36.7 [25.3, 50.2]	0.275	0.745	0.178	0.615
HCO_3_^−^(mmol/L)	11.1 [10.5, 13.7]	24.8 [19.0, 29.0]	21.4 [20.7, 25.4]	0.088	0.260	0.072	0.947
Base excess (mmol/L)	−23.4 [−24.4, −22.3]	−5.3 [−7.2, −4.3]	−1.3 [−4.7, −0.8]	0.008	0.043	0.024	0.363
Na (mmol/L)	151 [149, 154]	147 [145, 147]	144 [144, 145]	0.012	0.134	0.031	0.161
K (mmol/L)	9.1 [8.6, 9.6]	6.4 [5.5, 7.3]	5.0 [4.5, 5.0]	0.013	0.260	0.024	0.141
Ca (mmol/L)	1.34 [1.30, 1.37]	1.29 [1.21, 1.35]	1.27 [1.26, 1.29]	0.526	0.947	0.363	0.947
Cl (mmol/L)	123 [120, 124]	116 [112, 117]	113 [109, 114]	0.075	0.071	0.071	0.611
Anion gap (mmol/L)	27 [26, 27]	16 [10, 18]	14 [14]	0.030	0.175	0.022	0.906
Hematocrit (%)	40 [39, 41]	41 [41, 42]	42 [40, 42]	0.572	0.604	0.673	0.994
Hemoglobin (g/dL)	13.7 [13.2, 14.0]	14.0 [13.9, 14.1]	14.1 [13.7, 14.4]	0.650	0.679	0.742	0.976
Glucose (mg/dL)	226 [213, 227]	483 [299, 603]	416 [320, 515]	0.058	0.072	0.072	0.947
Lactate (mmol/L)	20.00 [19.90, 20.00]	4.80 [4.35, 7.88]	1.28 [0.75, 1.56]	0.005	0.022	0.022	0.260
Creatinine (mg/dL)	0.34 [0.32, 0.41]	0.39 [0.33, 0.51]	0.30 [0.30, 0.31]	0.153	0.804	0.287	0.196

Blood samples were collected from five mice per group. The 5% group showed lower base excess and higher lactate levels than the 0.6% group. In addition, they exhibited relatively high potassium levels. Each value is shown as the median and interquartile range

## Discussion

In this study, we investigated the effect of the concentration of volatile anesthetics that trigger MH on MH pathology. After exposure to sevoflurane, all mutant mice showed elevation of heart rate and body temperature and finally went into asystole. Also, they showed muscle rigidity, and these symptoms were considered derived from MH due to sevoflurane, based on clinical criteria [15]. Specifically, different concentrations of sevoflurane showed different patterns of MH development in the model mice. Although mice in the 0.6% group showed a longer time at maximum temperature and heart rate and a longer time to asystole, these mice showed a higher maximum temperature than the other groups and a higher heart rate than the 5% group. Notably, a high concentration of sevoflurane unexpectedly led to a lower body temperature and heart rate compared to low concentrations. Before these experiments, we expected that a higher concentration of volatile anesthetics might lead to the severe development of MH: more severe elevation of body temperature and heart rate. However, the mice exposed to 5% sevoflurane experienced cardiac arrest and died before their body temperature and heart rate increased significantly. A second experiment was conducted to explain these results.

According to our second experiment, base excess and lactate levels were much higher in the 5% group than in the 0.6% group, and potassium levels tended to be higher. These blood test results suggest that electrolyte abnormalities including hyperkalemia due to MH would be more severe with higher concentrations of sevoflurane than with lower concentrations. Hyperkalemia results from many factors, such as metabolic acidosis, renal dysfunction or rhabdomyolysis [16], and some of these factors might have affected severe hyperkalemia with higher concentrations of sevoflurane. The severity of MH is usually determined by the patient's body temperature [15, 17]; however, these animal experiments have shown that severe MH can occur with only a slight elevation in temperature when exposed to high concentrations of sevoflurane. The mechanism underlying these clinical courses remains unclear, but the sudden onset and severe development of hyperkalemia due to high concentrations of sevoflurane might lead to failure of circulation before heat caused by muscle hypermetabolism is moved into the core via circulation and heart rate increases responding hypermetabolic demands, and these factors may prevent the elevation of body temperature and heart rate. In clinical practice, the early symptoms of MH are often non-specific and diverse, making early detection challenging [18]. In fact, not all MH patients show fever as an initial symptom in clinical situations [19]. The underlying causes may be related to patient factors such as preoperative fever, physical activity, excitement, and psychological stress [20, 21]. Additionally, our findings suggest that the concentration of anesthetics may influence the diversity of symptoms in MH. In particular, in pediatric patients, volatile anesthetics are often used at higher concentrations during slow induction than during maintenance of anesthesia [22]. This could influence the epidemiological bias towards MH onset in younger individuals; however, further studies in clinical settings are needed to explore this potential relationship.

It is well known that intravenous anesthetics such as propofol are less likely to lead to MH than any volatile anesthetic [10, 23]. Therefore, total intravenous anesthesia (TIVA) should be administered to patients with a higher risk of MH [24, 25]. However, volatile anesthetics cannot be completely avoided in some cases, such as pediatric patients. To make matters worse, induction of anesthesia in children is performed using slow induction and relatively high concentrations of volatile anesthetics. Our study results demonstrated that high-concentration anesthesia induces fatal arrhythmia which subsequently leads to cardiac arrest without causing severe hyperthermia and accelerates the progression of symptoms after the onset of MH. Based on our findings, clinicians should be aware that in some cases, fatal arrhythmia may occur before the appearance of classic MH symptoms such as hyperthermia, especially under high-concentration volatile anesthesia. We cannot extrapolate our results by high-concentration anesthesia easily to clinical scenarios, because the clinical situations in which we administer volatile anesthetics to pediatric patients are not exactly similar to our study with prolonged exposure to high concentration of anesthetics. Nevertheless, clinicians should care about the necessity to minimize concentration of volatile anesthetics. These are key take-home messages from our study.

In contrast, mice administered with a lower concentration of sevoflurane showed prolonged development of MH in this study. MH mostly occurs immediately after exposure to triggering anesthetics, but there are some cases with delayed-onset MH [20, 26, 27]. Previous reports have shown that MH symptoms can appear within 1 h or later after exposure to volatile anesthetics [26–29]. Clinicians can lower the concentration of volatile anesthetics by using other anesthetics or analgesics like opioids [30], but according to our results, they should also care about the progression of MH at an unexpected time in such cases. We believe that when volatile anesthetics are used at low concentrations in balanced anesthesia, the onset or development of MH may be delayed.

Our research has some limitations. First, this study was conducted with a limited number of genetically modified mice, and a formal a priori power calculation was not performed. The number of animals was determined based on feasibility, the availability of the mutant strain, and sample sizes commonly reported in similar exploratory animal studies. As a result, statistical power may have been insufficient to detect certain between-group differences, including the absence of a statistically significant difference in the time to cardiac arrest between the 2.5% and 5% groups. However, other findings, including blood gas analysis, consistently demonstrated more severe metabolic acidosis, elevated lactate levels, and a tendency toward greater hyperkalemia in the high-concentration group. These results support the interpretation that higher concentrations of sevoflurane induced a more intense physiological response, despite the lack of statistical significance in cardiac arrest timing. Second, this study used only sevoflurane, and it is necessary to investigate other volatile anesthetics as well. In addition, elevated end-tidal CO₂ (EtCO₂) is often the first symptom of MH [6]; however, measuring EtCO₂ in mice is difficult, which may have limited the accurate assessment of MH onset in this study. Finally, arterial blood pressure was not measured in this study because continuous invasive monitoring was not available in our experimental setting. Therefore, we could not directly evaluate the potential contribution of hypotension to the progression toward asystole. Although anesthesia without surgical stimulation can induce hypotension, wild-type mice exposed to sevoflurane under identical conditions did not exhibit rapid deterioration of vital signs. Nevertheless, we cannot completely exclude the possibility that hypotension may have influenced the clinical course in mutant mice. The pathogenesis of MH remains unclear, and further studies using other anesthetics and additional animal experiments are needed.

In MH model mice, exposure to higher concentrations of sevoflurane accelerated progression of malignant hyperthermia. Interestingly, despite the rapid progression, the peak body temperature and heart rate were lower in the high-concentration group than in the low-concentration group. These findings suggest that rapid-onset and severe electrolyte disturbances triggered by high-concentration exposure may result in early cardiac arrest before typical peak values are reached.

## Data Availability

The datasets used and/or analyzed during the current study are available from the corresponding author on reasonable request.

## Abbreviations

MAC: Minimum alveolar concentration
MH: Malignant hyperthermia
TIVA: Total intravenous anesthesia
